# Immunometabolites Drive Bacterial Adaptation to the Airway

**DOI:** 10.3389/fimmu.2021.790574

**Published:** 2021-11-25

**Authors:** Kira L. Tomlinson, Alice S. Prince, Tania Wong Fok Lung

**Affiliations:** Department of Pediatrics, Vagelos College of Physicians & Surgeons, Columbia University, New York, NY, United States

**Keywords:** immunometabolism, host-pathogen interaction, bacterial persistence, metabolic adaptation, itaconate, succinate, *Staphylococcus aureus*, *Pseudomonas aeruginosa*

## Abstract

*Pseudomonas aeruginosa* and *Staphylococcus aureus* are both opportunistic pathogens that are frequently associated with chronic lung infections. While bacterial virulence determinants are critical in initiating infection, the metabolic flexibility of these bacteria promotes their persistence in the airway. Upon infection, these pathogens induce host immunometabolic reprogramming, resulting in an airway milieu replete with immune-signaling metabolites. These metabolites are often toxic to the bacteria and create a steep selection pressure for the emergence of bacterial isolates adapted for long-term survival in the inflamed lung. In this review, we discuss the main differences in the host immunometabolic response to *P. aeruginosa* and *S. aureus*, as well as how these pathogens alter their own metabolism to adapt to airway metabolites and cause persistent lung infections.

## Introduction

Healthcare-associated pneumonias, including those associated with COVID-19, are caused by opportunistic pathogens that readily adapt to the human airway. While the host immune response to lung pathogens has been extensively characterized (1, 2), less is known about how opportunistic bacteria survive in the lung despite hostile inflammatory conditions and appropriate antibiotic treatment.

Bacterial infection of the airway occurs in stages. Initially, environmental pathogens withstand the host immune response and antibiotic treatment by using virulence factors and acquired antimicrobial resistance genes to establish infection. Once a nidus of infection is formed, bacteria alter their metabolism and selectively regulate virulence to promote survival in the limited nutrient conditions and oxidative environment of the airway (3). Traditional experiments in microbial pathogenesis, including the use of defined deletion mutants and complemented strains, have been very effective in defining the virulence factors that are critical for establishing acute pneumonia (4, 5). However, the adaptations that enable bacteria to persist in the airway are not well understood.

To study adaptation of bacteria to the human airway, multiple research groups have used clinical strains from patients with chronic or persistent pneumonias, such as those with Cystic Fibrosis (CF) (6–8). The natural history of pulmonary infection in CF typically consists of initial infection with *Staphylococcus aureus*, followed by years of *S. aureus* and *Pseudomonas aeruginosa* co-infection, then predominant and intractable *P. aeruginosa* infection coinciding with increased pulmonary exacerbations and declining lung function (9). *S. aureus* and *P. aeruginosa* strains can thus be isolated from the same CF patient over years (10, 11). Genomic, transcriptional, and phenotypic data from these longitudinal isolates reveal the strategies that enable these common Gram-positive and Gram-negative pathogens to adapt to the airway for long-term survival. While the CF airway is usually polymicrobial, bacterial pneumonia, especially when attributed to the antibiotic-resistant ESKAPE pathogens, is often considered a single entity. This review will discuss the different strategies that *P. aeruginosa* and *S. aureus* use to survive in the human airway as well as the host factors that drive bacterial adaptation, with a particular focus on the roles of both host and bacterial metabolism.

## *P. aeruginosa* Induces a Succinate-Dominated Host Metabolic Reprogramming

The pathogenesis of acute *P. aeruginosa* infection often involves lipopolysaccharide (LPS)-displaying bacteria that activate host pattern recognition receptors (PRRs) like the Toll-like receptors (TLRs) (12). This bacterial recognition by host cells leads to downstream proinflammatory cytokine expression and phagocytic recruitment for bacterial clearance (13, 14). Recently, it has become increasingly appreciated that this inflammatory response is driven by changes in the metabolic activity of immune cells, a process called immunometabolism.

**Figure 1:**
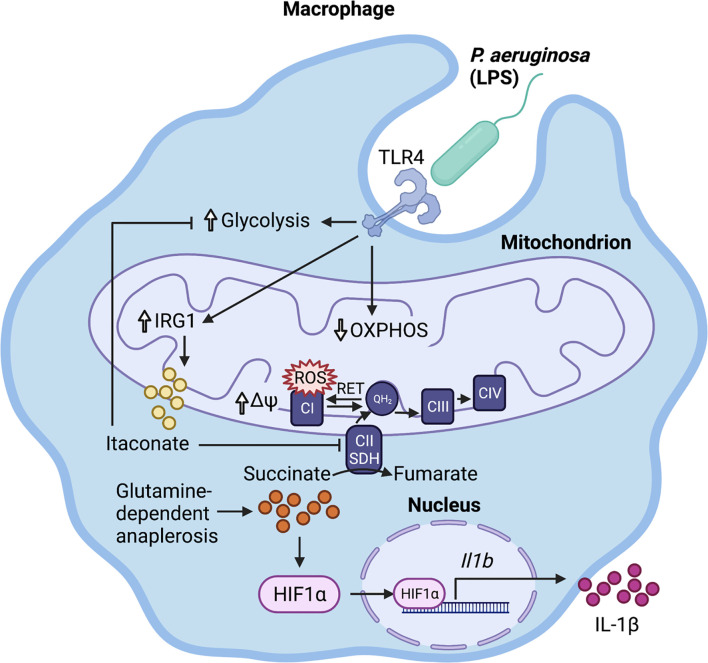
Immunometabolic response to *P. aeruginosa*. LPS activates the infected macrophage, stimulating glycolysis and downregulating oxidative phosphorylation (OXPHOS). This promotes glutamine-dependent anaplerosis, which replenishes succinate for oxidation to fumarate by mitochondrial succinate dehydrogenase (SDH). The resulting increase in mitochondrial transmembrane potential (∆Ψ) and over-reduction of the ubiquinone pool reverse electron flow to complex I, where they escape as reactive oxygen species (ROS). Both ROS and succinate stabilize HIF-1α, which translocates to the nucleus and binds to its target promoter regions, increasing *Il-1β* transcription. The succinate-driven inflammation is regulated by the production of the anti-inflammatory metabolite itaconate.

In response to LPS, macrophages become activated through TLR4 signaling and undergo metabolic reprogramming (15). This comprises upregulation of aerobic glycolysis and downregulation of oxidative phosphorylation (OXPHOS) to meet the cell’s energy requirements (**Figure 1**). While this metabolic shift may seem counterintuitive given that OXPHOS is more energy efficient (36 molecules of ATP/glucose molecule), aerobic glycolysis can generate ATP (2 molecules of ATP/glucose molecule) faster than OXPHOS, akin to the Warburg effect in cancer cells (15, 16). These metabolic changes are accompanied by increased production of the metabolite succinate through glutamine-dependent anaplerosis and the gamma-aminobutyric acid (GABA)-shunt (17) (**Figure 1**). Increased mitochondrial oxidation of succinate to fumarate *via* succinate dehydrogenase (SDH/respiratory complex II) and the resulting elevation of mitochondrial membrane potential (Ψ) drive the production of reactive oxygen species (ROS) *via* reverse electron transport (RET) (18) (**Figure 1**). Succinate and ROS stabilize the host transcription factor hypoxia-inducible factor 1α (HIF-1α) by inhibition of prolyl hydroxylase (PHD) in the cytosol (19) (**Figure 1**). This causes an increase in the transcription of genes encoding glycolytic enzymes and the proinflammatory cytokine IL-1β, which is expected to promote bacterial clearance.

It is important to note that, in addition to fueling succinate production, glutamine-dependent anaplerosis can also increase α-ketoglutarate levels. α-ketoglutarate promotes anti-inflammatory pathways through epigenetic changes [reviewed in (20)]. Therefore, glutaminolysis can drive both pro-inflammatory and anti-inflammatory metabolic programs, and the ratio of succinate to α-ketoglutarate is critical for determining macrophage polarization (21).

While these metabolic changes have been extensively detailed in macrophages responding to LPS *in vitro*, infection with live *P. aeruginosa* also results in increased succinate accumulation and production of IL-1β in the murine lung (22). Of note, increased succinate production is an inherent property of the CF lung, even in the absence of infection (22), due to a lack of sufficient membrane-bound CF transmembrane conductance regulator (CFTR) and impaired activity of the metabolic regulator Phosphatase and Tensin Homolog deleted on Chromosome 10 (PTEN) (22, 23).

Increases in proinflammatory cytokines do not always clear pathogens. This is clearly exemplified by the current COVID-19 pandemic, whereby SARS-CoV2-induced cytokine storm results in excessive inflammation that fails to clear the viral pathogen and instead contributes to immunopathology and mortality (24, 25). *P. aeruginosa* also stimulates IL-1β production by activating the NLRC4 inflammasome in alveolar macrophages, which enhances bacterial infection in a murine pneumonia model (26). Dampening inflammasome activation or inhibiting IL-1β signaling *via* the use of *Il-1r* or *Il-18r* null mice promotes bacterial clearance and reduces immunopathology (26).

In order to restore homeostatic balance and counteract succinate-driven inflammation, myeloid cells upregulate the expression of Immune Responsive Gene 1 (*Irg1*/*Acod1*) to produce the immunometabolite itaconate (**Figure 1**). Interestingly, this dicarboxylate, which structurally resembles succinate, dampens inflammation *via* its effect on several host pathways described below. Itaconate is abundantly produced in the host airway during *P. aeruginosa* infection as well as in the CF airway (22, 27).

## Itaconate Counteracts Succinate-Driven Inflammation

Although itaconate was originally discovered in 1836 (28), its immunoregulatory function was only elucidated in 2016 (29, 30). Using *Irg1* knockout cells and exogenous itaconate, multiple groups found that itaconate inhibited SDH, thus preventing succinate oxidation, ROS production *via* RET, HIF-1α stabilization, and IL-1β production (29–31) (**Table 1**). Further investigations have utilized derivatives like 4-octyl-itaconate (4OI) and dimethyl-itaconate (DMI) to show that itaconate mitigates inflammation by modifying cysteine residues, inhibiting glycolytic enzymes that energetically sustain immune cell activation, preventing NLRP3 inflammasome activation, and activating anti-inflammatory and anti-oxidant pathways by promoting ATF3 and NRF2 activation (32, 33, 36–38, 40, 41) (**Table 1**). These pathways subsequently drive macrophage polarization. For example, itaconate plays a key role in IL-33-induced alternatively activated macrophages (AAMs) by promoting GATA3 expression (42). Itaconate and its derivatives remain the focus of ongoing investigation, even as a potential therapeutic agent to treat COVID-19 (43), given their role in the control of other viruses such as Zika (44) and influenza (45).

**Table 1:** Mechanisms of action of itaconate, and its derivatives.

Confirmed	Contested	Immune responsive gene1 (*Irg1*)	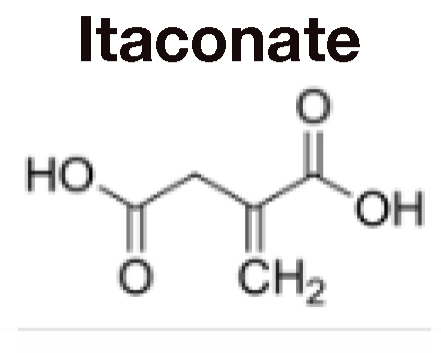	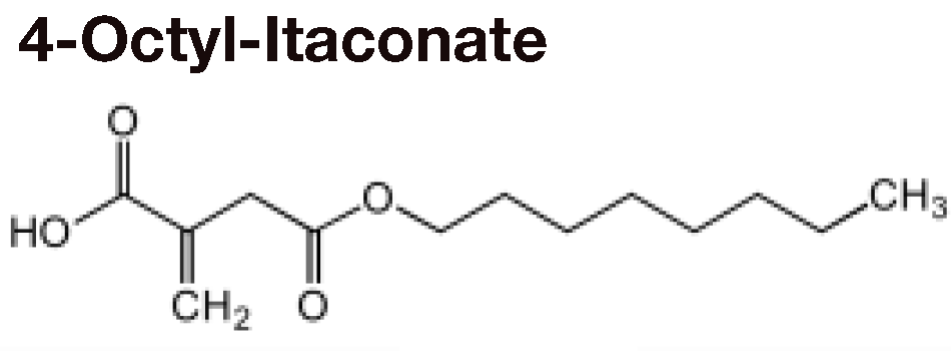		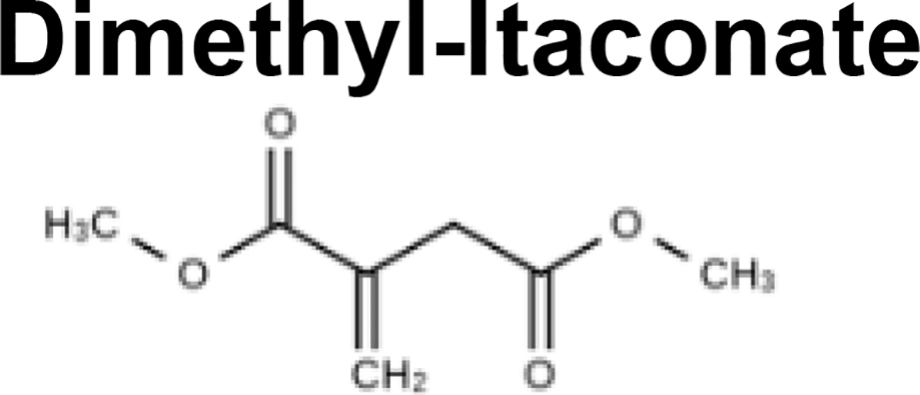	
		
**Modifies cysteine residues and inhibits metabolic enzymes**			(31)	(32, 33)			
**Reduces glucose consumption and lactate production**		(31)	(31)	(32)		(30)	
**Inhibits SDH and induces succinate accumulation**		(29, 30, 34)	(29, 30, 34)	(34)		(30)	(34)
**Suppresses IL-1β, IL-6, IL-12, IL-18 production**		(30, 35)	(31, 34)	(32, 33, 34, 36)	(36) (high doses)	(30, 34, 36)	(36) (high doses)
**Promotes IFN-β release and Type I IFN signaling**		(34)	(34)	(33, 34)		(34)	
**Limits NLRP3 inflammasome activation**		(37, 38)	(37)	(34)		(30)	
**Promotes OXPHOS and PPP-driven ROS production**		(35, 39)	(35, 40)				
**Alkylates KEAP1 and activates Nrf2 signaling**			(34)	(33, 34, 41)		(34, 40)	
**Reduces IκB levels *via* ATF3**			(34)	(34)		(34, 40)	

Recently, some of the mechanisms of action initially ascribed to itaconate are being reevaluated in light of major differences in the effects of itaconate versus its derivatives (34) (**Table 1**). DMI and 4OI were originally used because of their cell permeability, but multiple studies have shown that itaconate also reaches the cytoplasm when administered exogenously to cells (34, 37). Direct comparisons of itaconate and its derivatives have revealed that DMI and 4OI are more electrophilic than itaconate and, subsequently, target different processes within mammalian cells. While DMI and 4OI are able to activate ATF3 and NRF2 signaling, itaconate does not (34) (**Table 1**). Additionally, itaconate promotes Type I interferon signaling, while its derivatives suppress IFN-β production (34) (**Table 1**). Despite these differences, itaconate and its derivatives share some core mechanisms of action, including inhibition of glycolysis, inhibition of succinate oxidation, suppression of pro-inflammatory cytokine release, inhibition of the NLRP3 inflammasome, and promotion of ROS generation through OXPHOS and the pentose phosphate pathway (PPP) (**Table 1**). Overall, itaconate regulates inflammation by modulating the metabolic reprogramming that enables immune cells to release cytokines and antibacterial factors into the surrounding tissue.

## Succinate and Itaconate Fuel *P. aeruginosa* Lung Infection

**Figure 2:**
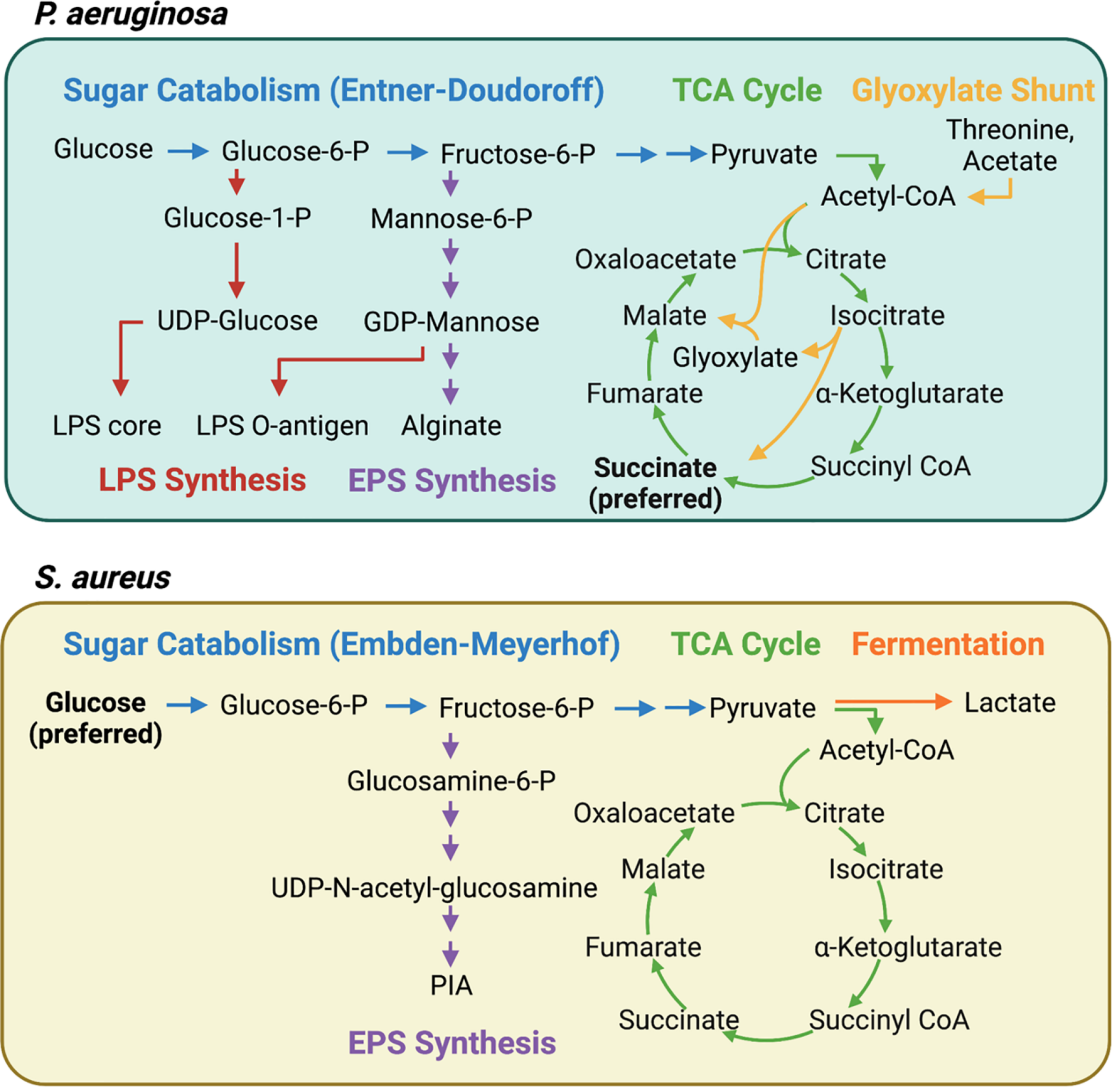
Interconnected pathways contributing to central carbon metabolism, LPS, and EPS synthesis in *P. aeruginosa* and *S. aureus*. *P. aeruginosa* and *S. aureus* have distinct carbon preferences and metabolic pathways that contribute to pathogenesis. Carbon catabolite repression (CCR) ensures that *P. aeruginosa* consumes succinate until it is depleted whereas *S. aureus* preferentially consumes glucose. The continuous consumption of succinate by *P. aeruginosa* generates endogenous bacterial reactive oxygen species (ROS) *via* increased aerobic respiration. *P. aeruginosa* adapts by bypassing OXPHOS and upregulating the glyoxylate shunt and the Entner-Doudoroff pathways, resulting in increased extracellular polysaccharide (EPS) synthesis. *S. aureus*, meanwhile, is highly dependent on glycolysis and fermentative metabolism for survival during infection. When glycolysis is interrupted, as it is in itaconate-rich environments, carbon is shunted into EPS synthesis.

As host cells undergo immunometabolic reprogramming and release metabolites, cytokines, and antimicrobial factors into the lung environment, *P. aeruginosa* adapts through its own metabolic flexibility. This metabolic versatility is conferred by a global regulatory system termed carbon catabolite repression (CCR) that coordinates the assimilation of a preferred compound over other potential carbon sources (46). CCR in *Pseudomonas* is significantly different from that in *Firmicutes* or even in the *Enterobacteriaceae*. For example, the preferred carbon sources for *P. aeruginosa* are organic acids, particularly succinate, whereas *S. aureus* and *E. coli* preferentially consume glucose over other carbon sources [reviewed in (46)] (**Figure 2**). Therefore, in a setting replete with succinate, such as the inflamed airway, *P. aeruginosa* would benefit from the abundant supply of its preferred carbon source.

**Figure 3:**
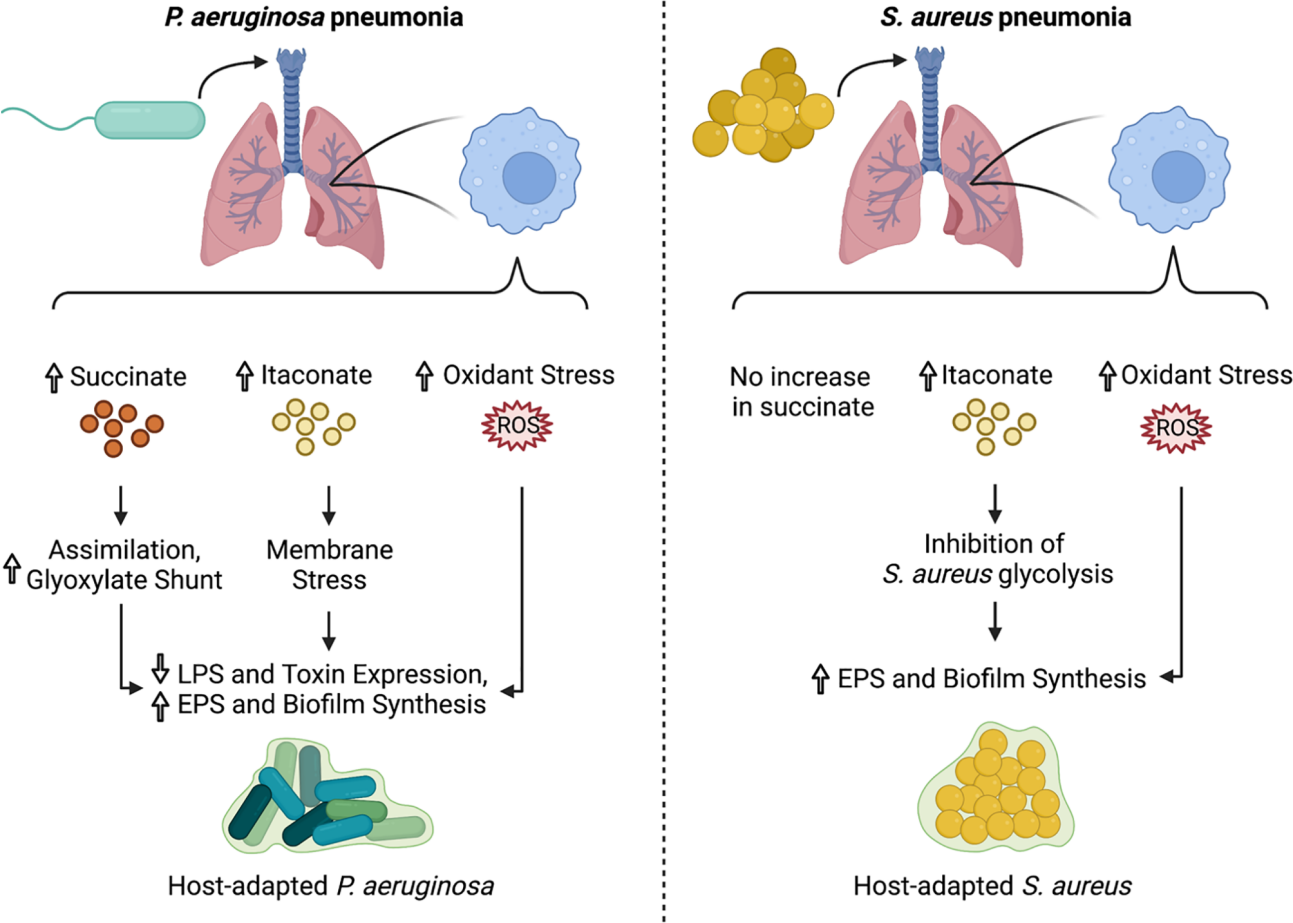
Impact of immunometabolites on *P. aeruginosa* and *S. aureus* adaptation to the lung microenvironment. During infection with *P. aeruginosa*, airway immune cells produce succinate, itaconate, and ROS. This creates a strong selective pressure for isolates that adapt *via* metabolic rewiring, decreased LPS surface display and toxin production, and increased biofilm synthesis. In contrast, *S. aureus* infection induces accumulation of itaconate and ROS, but not succinate in the airway. Both itaconate and ROS select for metabolically-altered *S. aureus* strains that exhibit decreased glycolytic activity and increased EPS and biofilm production.

*P. aeruginosa* grown in a high succinate concentration *in vitro* induced more inflammation and succinate release *in vivo*, and exhibited increased colonization of the murine airway (22). However, the continuous assimilation of succinate by *P. aeruginosa*, as is the case in the CF airway, results in increased bacterial production of endogenous ROS *via* aerobic respiration, providing a steep selective pressure for ROS-adapted isolates. These successful isolates increase metabolic flux through the glyoxylate shunt and the Entner-Doudoroff pathway, bypassing aerobic respiration to produce extracellular polysaccharides (EPS) such as alginate, which are used to produce biofilms and shield the bacteria from oxidant stress (22) (**Figure 2**). Exogenous ROS from activated phagocytes also contribute to the selection of these host-adapted isolates, which are characterized by decreased LPS at their surface, reduced toxin production, and increased biofilm synthesis (22, 27) (**Figure 3**). Of note, increased EPS surface display by these isolates promotes more itaconate production, creating a positive feedback loop that drives intractable infection (22, 27).

Itaconate is toxic to many bacterial species because it inhibits isocitrate lyase (AceA), a key enzyme of the glyoxylate shunt, which is required for bacterial survival *in vivo* (47–54). *P. aeruginosa* harbors three genes, namely *ict*, *ich* and *ccl*, that enable it to catabolize itaconate and produce the energetic intermediates acetyl-coA and pyruvate (27, 55). Clinical isolates from CF airways prefer to use itaconate over succinate as a carbon source and, unlike the laboratory strain PAO1, are impaired in their ability to infect *Irg1^-/-^* mice (27), exemplifying how *in vivo* adaptation alters both the metabolic preferences and immunostimulatory capacity of these Gram-negative bacteria. Itaconate thus serves as a key metabolic signal that works in concert with other metabolites, like succinate, to promote *P. aeruginosa* adaptation to the airway.

## *S. aureus* Induces an Itaconate-Dominated Immunometabolic Response

As a Gram-positive pathogen lacking LPS, *S. aureus* induces a distinct immunometabolic response from the one detailed above for *P. aeruginosa*. During *S. aureus* infection, itaconate, but not succinate, accumulates in the airway (22, 56) (**Figure 3**). This itaconate-dominated response is accompanied by a distinct cytokine profile, characterized by diminished levels of some pro-inflammatory cytokines, like IL-1β and IL-6, but not others, like TNFα (56). The selective reduction in IL-1β and IL-6 is likely due to the inability of *S. aureus* to stimulate TLR4-driven succinate accumulation and HIF-1α stabilization.

The host immune response to *S. aureus* infection depends on TLR2, which responds to bacterial cell surface and secreted factors like lipopeptides (57). TLR2 plays a prominent role in the immune response to early staphylococcal colonization of the lung, but is not essential for bacterial clearance and host survival during pneumonia (58), suggesting that there are other pathways that the host uses to sense and respond to *S. aureus* during lung infection. This redundancy is important, given that *S. aureus* can produce super antigens that bind and inactivate TLR2 (59).

Though IL-1β and IL-6 are less abundant in the *S. aureus-*infected airway, they still play important roles in infection outcomes. As in *P. aeruginosa* lung infections, IL-1β does not facilitate bacterial clearance and instead exacerbates tissue damage during *S. aureus* pneumonia. Mice lacking the interferon receptor *Ifnlr1* demonstrate reduced IL-1β production along with improved bacterial clearance during *S. aureus* lung infection, and administration of recombinant IL-1β to these mice worsens bacterial burden (60). During *S. aureus* lung infections, IL-1β is generated *via* inflammasome-dependent and independent mechanisms, including neutrophil elastase (60). These alternative mechanisms likely sustain IL-1β production during chronic staphylococcal infections, given that inflammasome-driven inflammation and tissue damage is dependent on *S. aureus* alpha toxin (Hla), which is often downregulated in clinical strains (56, 61). IL-6, meanwhile, modulates lung inflammation through mechanisms that depend on the nature of the stimulus and the severity of inflammation, promoting neutrophil infiltration in response to staphylococcal peptidoglycan but limiting neutrophil infiltration, cytokine production, and tissue damage in response to staphylococcal lipoteichoic acid (62).

The role of metabolites in regulating inflammation during *S. aureus* lung infection has not yet been defined. The majority of the studies that delineated the interplay between inflammation and metabolism used LPS as a stimulus, and thus involved TLR4-related pathways that may not be relevant to *S. aureus* infection. Given that LPS is often used to stimulate immunometabolic reprogramming, another major question that remains is how *S. aureus* induces itaconate production. A recent study of the role of itaconate in *S. aureus* endophthalmitis indicates that TLR2 signaling is not sufficient, as heat-killed *S. aureus*, lipoteichoic acid, and peptidoglycan do not stimulate IRG1 expression to the same extent as live bacteria (63). Instead, IRG1 expression can be mitigated by administration of a mitochondrial ROS scavenger, suggesting that host mitochondrial stress and/or oxidants promote IRG1 expression during *S. aureus* infection (56). Mitochondrial ROS production and itaconate accumulation in the murine airway depend on *S. aureus* glycolysis, demonstrating that bacterial metabolism itself stimulates itaconate generation during *S. aureus* lung infection (56). The exact mechanism that connects bacterial metabolism to host metabolic reprogramming still needs to be determined.

## Itaconate Drives Persistent *S. aureus* Lung Infection

Just as *S. aureus* stimulates a distinct immunometabolic response when compared with *P. aeruginosa*, it also employs different strategies for adaptation to the human airway. As mentioned above, *S. aureus* preferentially consumes glucose, and its glucose metabolism is intricately linked with toxin and extracellular polysaccharide synthesis through carbon catabolite repression, regulated by CcpA and CodY [reviewed in (64)]. Increased glucose consumption can also be used to fuel lactate fermentation, which enables the bacteria to maintain redox balance within the cell in the setting of oxidant stress (65). As such, glucose consumption is critical for survival during inflammation, and mutants that are unable to transport glucose into the cell or metabolize it *via* glycolysis are impaired in skin, soft tissue, and lung infections (56, 65–67).

Unlike *P. aeruginosa*, *S. aureus* is unable to catabolize itaconate, and itaconate inhibits *S. aureus* growth in activated host immune cells (53). Instead of targeting the glyoxylate shunt, which is absent in *S. aureus*, itaconate inhibits staphylococcal glycolysis (Embden-Meyerhof), mirroring one of its mechanisms of action in mammalian cells (56). This glycolytic inhibition rewires bacterial metabolism to promote carbon flux through upstream pathways that synthesize extracellular polysaccharides used in biofilms (56) (**Figure 2**). As such, longitudinal isolates that represent adaption to the CF lung over 15 years exhibited increased biofilm production in the presence of itaconate (56) (**Figure 3**). This differs from the mechanism by which itaconate promotes *P. aeruginosa* biofilms, which involves inducing membrane stress to downregulate LPS production and promote EPS synthesis (27). Nevertheless, these adaptive mechanisms converge on biofilm formation, which is beneficial not just to the microorganism as protection from phagocytosis and antimicrobial factors, but also to the bacterial community, which includes both *S. aureus* and *P. aeruginosa* during chronic lung infections.

While *S. aureus* does not induce succinate accumulation, it is often in succinate rich environments, particularly during co-infection with *P. aeruginosa* in the CF airway. In contrast to its effects on *P. aeruginosa*, succinate inhibits *S. aureus* growth and biofilm production in a dose-dependent manner, likely by inhibiting consumption of its preferred carbon sources, including glucose (22). Accordingly, *S. aureus* cultured in high succinate concentrations is impaired in its ability to colonize the airway and lungs of mice, unlike *P. aeruginosa* grown in the presence of high succinate concentrations (22). These studies reaffirm that common airway pathogens have different metabolic preferences that are tailored to the immunometabolic response they induce in the host.

## Concluding Remarks

In recent years, it has become increasingly apparent that immunometabolism plays a key role in the pathogenesis of infections. Microbial metabolism has often been neglected in these studies, with many research groups instead turning to the use of LPS as a standard proxy for bacterial stimulation. There is increasing evidence, however, that microbial metabolic flexibility is not only critical for bacterial persistence during infection, but also in shaping the host immunometabolic response (68–70). Importantly, this metabolic interplay between host and pathogen varies by microbe and infection site. Future investigations into the immunometabolism of infection should continue to include diverse pathogens and tissues, taking care to address the differences in host response to active microbial metabolism versus inert PAMPs. These studies should also address the systemic consequences of immunometabolism, as comparatively little is known about the activity of metabolites that are absorbed into the bloodstream during infection.

While we have focused on the roles of just a few metabolites in driving bacterial adaptation, other airway metabolites produced during infection are likely to influence host-pathogen metabolic interactions and infection outcomes. For example, analysis of sputum samples from CF and non-CF patients revealed higher amino acid concentrations in CF sputum (71). This is particularly interesting given that both *P. aeruginosa* and *S. aureus* consume amino acids, with a hierarchical preference for some amino acids over others (72) [reviewed in (73)]. Conversely, amino acid starvation in many pathogenic bacteria induces virulence gene expression. Thus, it is tempting to speculate that the abundance of amino acids in the CF airway progressively leads to decreased virulence gene expression as a result of increased amino acid consumption.

Other factors that may influence the metabolic interaction between the host and pathogen include the contribution of microbiota-derived metabolites or metabolites generated by co-infecting pathogens. Microbiome-derived metabolites such as short chain fatty acids have been shown to regulate the function of key immune cells, including CD8^+^ T cells and their ability to recall infection (74). During co-infection of the CF lung, *S. aureus* produces acetoin, which is used as a carbon source by *P. aeruginosa*, decreasing the toxic effect of accumulated acetoin on *S. aureus* and promoting the persistence of both bacteria (75). These studies highlight the importance of considering the metabolites originating from organisms other than the host and pathogen of interest when studying the metabolic underpinnings of infection pathogenesis.

Microbial adaptation to the host can be difficult to study because animal models of chronic pulmonary infection are limited. This problem is often circumvented by using clinical isolates that have adapted to the human airway. One caveat to this approach is that these metabolic adaptations may not be maintained over time as the isolates are repeatedly exposed to artificial *in vitro* conditions that do not adequately mimic the tissue microenvironment. Altogether, while targeting host immunometabolism as an alternative or complementary therapeutic strategy to enhance the clearance of multidrug-resistant bacteria is an alluring prospect, host-pathogen metabolic interactions and additional factors that influence these dynamics require further study.

## Author Contributions

KLT, ASP, and TWFL wrote and edited the manuscript. All authors have read and agreed to the published version of the manuscript.

## Funding

ASP was supported by NIH grant 1R35HL 135800 and the Cystic Fibrosis Foundation CFF PRINCE18G0.

## Conflict of Interest

The authors declare that the research was conducted in the absence of any commercial or financial relationships that could be construed as a potential conflict of interest.

## Publisher’s Note

All claims expressed in this article are solely those of the authors and do not necessarily represent those of their affiliated organizations, or those of the publisher, the editors and the reviewers. Any product that may be evaluated in this article, or claim that may be made by its manufacturer, is not guaranteed or endorsed by the publisher.
